# Endoscopic Ultrasound as a Diagnostic Tool for the Mediastinum and Thorax

**DOI:** 10.3390/jcm14144836

**Published:** 2025-07-08

**Authors:** Sara Nikolic, Lucía Guilabert, Giuseppe Vanella, Catalina Vladut, Giuseppe La Mattina, Giuseppe Infantino, Elio D’Amore, Cecilie Siggaard Knoph, Giacomo Emanuele Maria Rizzo

**Affiliations:** 1 Department of Gastroenterology, University Medical Center Maribor, 2000 Maribor, Slovenia; 2 Gastroenterology Department, Dr. Balmis General University Hospital, Instituto de Investigación Sanitaria y Biomédica de Alicante (ISABIAL), 03010 Alicante, Spain; 3 Division of Pancreato-Biliary Endoscopy and Endosonography, Pancreas Translational and Clinical Research Center, IRCCS San Raffaele Scientific Institute, Vita-Salute San Raffaele University, 20132 Milan, Italy; 4 Department of Gastroenterology, “Prof. Dr. Agrippa Ionescu” Clinical Emergency Hospital, 011356 Bucharest, Romania; 5 University of Medicine and Pharmacy “Carol Davila”, 050474 Bucharest, Romania; 6 Broncopneumologia Interventistica, AOOR Villa Sofia Cervello, 90127 Palermo, Italy; 7 Gastroenterology and Endoscopy Unit, Istituto Mediterraneo per i Trapianti e Terapie ad Alta Specializzazione, IRCCS—ISMETT, 90127 Palermo, Italy; 8 Centre for Pancreatic Diseases, Department of Gastroenterology & Hepatology, Aalborg University Hospital, 9100 Aalborg, Denmark

**Keywords:** EUS, EBUS, bronchoscopy, endoscopy, lung masses, mediastinum

## Abstract

Endoscopic ultrasound (EUS) is a helpful tool for the study of the mediastinum, a challenging region for both transesophageal and endobronchial (EBUS) endosonography. This area is divided into sections and contains numerous lymph nodes essential for the staging and diagnosis of conditions like lung cancer, sarcoidosis, and infections. EUS allows for detailed examination of the mediastinal region, identifying various kinds of abnormalities, whether they are benign cysts or malignant tumors. The aim of this narrative review is to provide a clear overview of how EUS contributes to mediastinal diagnostics and to offer practical insights for clinicians. A comprehensive, non-systematic search of PubMed was conducted by the authors to identify relevant studies. EUS methods, such as elastography and contrast-enhanced imaging, have improved diagnosis by analyzing tissue stiffness and blood flow, and they help endosonographers distinguish between different conditions. EUS-guided tissue sampling techniques, like fine needle aspiration and biopsy, are crucial for detecting cancer and examining lymph nodes in a minimally invasive way. By combining EUS with endobronchial ultrasound, operators can achieve more accurate results, especially in cancer staging and treatment planning. Overall, this approach is a key tool in treating thoracic and mediastinal conditions.

## 1. Introduction

Even for experienced clinicians, the mediastinum is an enigmatic anatomical region, home to vital structures that are not easily accessible for histological assessment. Accurate evaluation of this space, positioned between the lungs, is essential, particularly for investigating primary and secondary abnormalities, including abnormal lymph nodes (LNs). Endosonography of the thorax and mediastinum presents unique challenges due to its intricate anatomy and limited accessibility. In this review, we examine the current role and potential of linear endoscopic ultrasound (EUS) in mediastinal assessment, highlighting established clinical applications and including the evaluation with endobronchial ultrasound (EBUS). Our goal is to provide a clear overview of how EUS contributes to mediastinal diagnostics and to offer practical insights for clinicians involved in managing thoracic and upper gastrointestinal diseases.

## 2. Methods

This is a narrative review aiming to critically synthesize current evidence on the diagnostic application of EUS as well as endobronchial EUS (EBUS) in the evaluation of the mediastinum and thorax. A comprehensive, non-systematic search of PubMed was conducted by the authors to identify relevant original studies, clinical guidelines, expert opinions, and meta-analyses published up to April 2025. Emphasis was placed on literature addressing anatomical, technical, and diagnostic findings of these procedures.

## 3. Results

### 3.1. Thorax and Mediastinal Lymph Nodes Stations

Mediastinum contains multiple organs and vessels and plays a central role in lymphatic drainage. It can be divided into three functional compartments: the anterior (pre-vascular), middle (visceral), and posterior (paravertebral) mediastinum. There are LNs in all three functional compartments, although most of them are located in the anterior and middle ones. Compartmentalizing the mediastinum aids in narrowing the broad range of differential diagnoses for thoracic conditions including, but not limited to infections like tuberculosis, the nodal spread of lung cancer, sarcoidosis, lymphoma, silicosis, and asbestosis. According to the lymph nodes map of the International Association for the Study of Lung Cancer (IASLC) [1,2], we can divide intrathoracic LNs in stations and zones [3], especially when approaching by EBUS evaluation, as reported in Table 1.

### 3.2. Lung, Thyroid and Other Mediastinal Lesions

Diseases of the lung, thyroid, and other mediastinal organs can include tumors, cysts, and other abnormalities. These lesions can be benign or cancerous, and they could be studied by EUS depending on their location in the mediastinum. Thyroid nodules and masses are usually studied by conventional ultrasound, even if EUS could also be able to evaluate the thyroid. Mediastinal lesions can be thymoma, lymphoma, germ cell tumors (rare tumors that are usually benign), parathyroid adenomas, neurogenic tumors (commonly located in the posterior mediastinum), and cysts.

### 3.3. EUS Anatomical Evaluation

**Figure 1:**
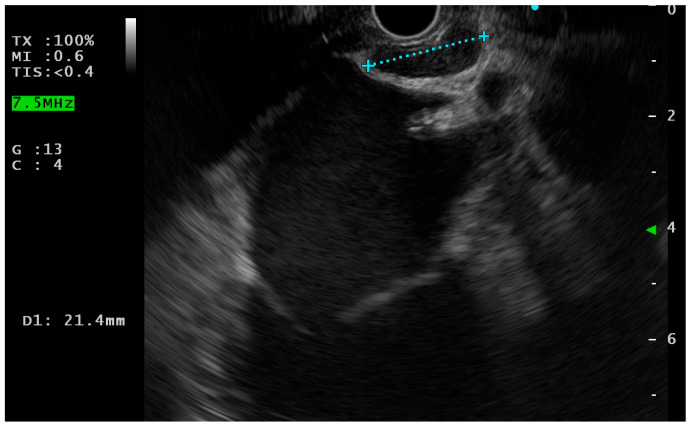
Endoscopic ultrasound (EUS) view of subcarinal space with a lymph node (21.4 mm of diameter).

The two most important mediastinal LNs stations to evaluate when performing digestive (transoesophageal) EUS are the subcarinal space (station 7) and the aorto-pulmonary window (station 4L/5), since they are the most accessible through the transesophageal approach [4]. The anatomical evaluation of mediastinum starts in the distal esophagus (35 to 40 cm on the scope shaft) at the gastroesophageal junction; here, the scope can be torqued clockwise and counterclockwise visualizing either the aorta or the liver and then we have to torque the scope (clockwise or anti-clockwise), looking for the heart [5]. Here, if we withdraw the scope, the left atrium comes into view. From this point, if you continue to slightly withdraw the scope, the left atrium is positioned in the left part of the screen and the pulmonary artery comes into the view on the right part of the screen. With a linear scope, subcarinal space is defined as the space between the pulmonary artery (on the right) and the left atrium (on the left) (Figure 1).

**Figure 2:**
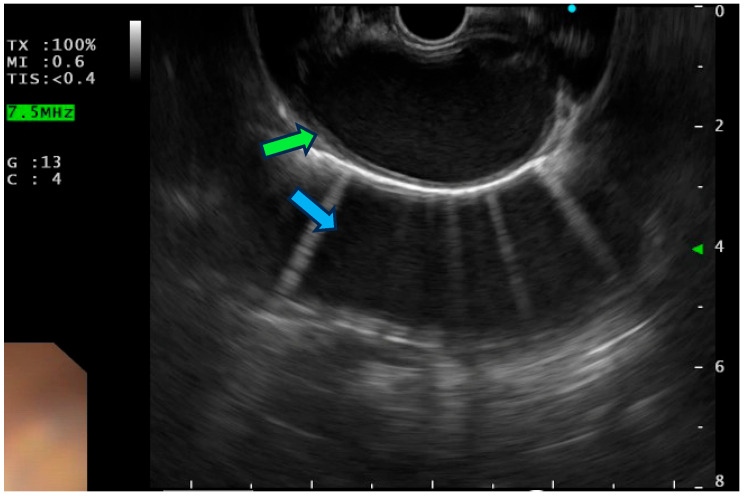
EUS view of the mediastinal side of thoracic aorta (green arrow) with mirror image of the aorta (blue arrow).

Once we finished studying the subcarinal space (looking for LNs of station 7), we have to return back to the aorta. From here, we start the part of the examination for reaching the aorto-pulmonary window. Starting from the aorta, where we will see a mirror image of the descending portion (Figure 2), we withdrew the scope until we found the aortic arch.

**Figure 3:**
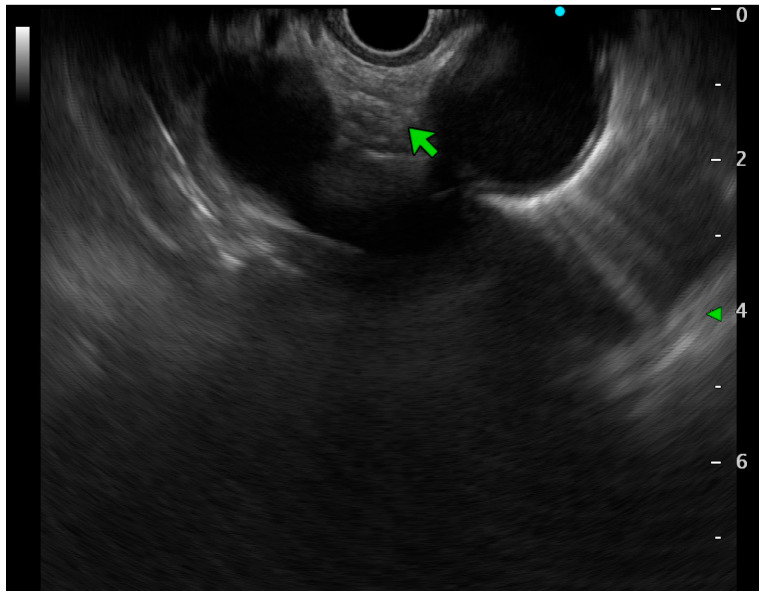
EUS view of the aorto-pulmonary window, between the aorta (right side) and pulmonary artery (left side) (green arrow indicates where lymph nodes could be identified).

From here, you have to torque the scope clockwise to about 60 degrees and tip “up”, so you will reach the aortopulmonary window, defined as the space between the aorta (on the right side of the screen) and the pulmonary artery (on the left part of the screen, Figure 3). From this window, station 4L, 5, and 6 can be visualized, even if only 4L and 5 are approachable for tissue acquisition (TA) [6].

### 3.4. EBUS Anatomical Evaluation

**Figure 4:**
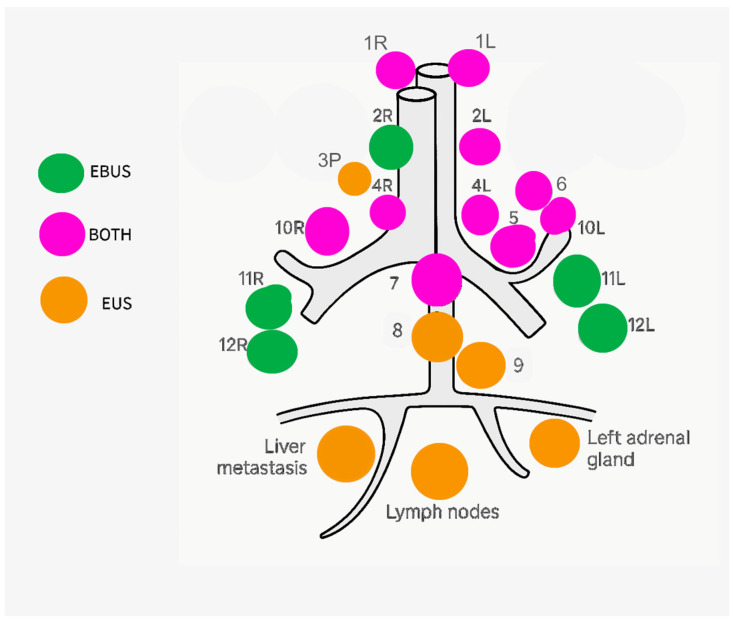
Endoscopic ultrasonography (EUS) and endobronchial ultrasonography (EBUS) in the evaluation of mediastinal LNs stations and other stations related to thoracic cancers.

In the case of the endobronchial approach (EBUS), when LNs in station 1 (1L and 1R) are enlarged and close to the trachea, they may be visible within the first centimeter below the vocal cords. Usually, they are not suitable for transbronchial needle aspiration (TBNA) for the conformation of trachea, even when they are enlarged, they become identifiable, so they represent an N3 station in Non-Small Cell Lung Cancer (NSCLC) staging. Station 2R can be visualized in the upper third of the trachea. To identify its lower boundary, it is useful to first locate station 2L, which lies on the left lateral wall of the trachea, near the left jugular vein and common carotid artery. Then, by rotating the bronchoscope to the right at the same cranio-caudal level, station 2R can be found. Station 3, divided into anterior (prevascular, 3A) and posterior (retrotracheal, 3P) compartments, is rarely accessible but may be differently reached with EBUS (3A) and EUS (3P). Station 4 is best explored by positioning the transducer at 1–2 o’clock in the 2nd–4th intercartilaginous space above the carina. Specifically, station 4R lies anterior to the superior vena cava, so, starting with the visualization of the azygos vein (the lower margin of this station), the area above this vein can be explored, allowing identification of station 4R. These nodes may extend in front of the trachea and are sometimes referred to as pre-carinal nodes. Differentiating station 4R from station 10R is critical in NSCLC staging, and the azygos vein is the key landmark for this. On the other hand, station 4L is located just above the origin of the left main bronchus, between the aorta and pulmonary artery. It can also be visualized using a transesophageal approach (EUS or EUS-B) (Figure 4).

Station 5 lies just distal to 4L, but still at a similar level. It is challenging to sample due to the interposition of the pulmonary artery and aorta. However, EUS provides a wider acoustic window and better needle control. Station 6, situated lateral to the ascending aorta and arch, can be reached from the same position used for 4L and 5, or slightly more cranially. Due to the interposition of the aorta, sampling of station 6 is not feasible. Station 7 is located beneath the carina and can be approached from either the right or left main bronchus. Its identification is primarily based on endoscopic anatomy rather than ultrasound landmarks, and it is easily accessible via either EUS or EBUS. Stations 8 and 9 are better approached by transesophageal EUS. Station 10R can be seen by placing the transducer anterior to the right main bronchus at 12–1 o’clock, in the first or second intercartilaginous space below the carina. It lies just behind the right pulmonary artery. The azygos vein is essential to differentiate this from station 4R, as abovementioned. Station 11 lies between the upper and intermediate bronchi. To view it, the probe should be positioned below the spur separating these branches. Hilar vessels could limit sampling at this station, even if the advantage of ultrasound view permits a real time assessment of the anatomy while sampling, increasing the probability to avoid vessels and be safe. In addition, on the right side of station 11 it is possible to distinguish between sampling 11Rs LNs (transducer between right upper bronchus and intermediate bronchus) and 11Ri LNs (transducer between middle bronchus and inferior bronchus).

### 3.5. EBUS Applications

Systematic mediastinal staging of lung cancer is crucial to identify patients eligible for surgery. Particularly, the assessment of mediastinal involvement is the primary prognostic factor when distant metastases are absent [7,8]. Therefore, mediastinoscopy, mediastinotomy or video-assisted thoracoscopic surgery have been used as staging techniques. The accuracy of these invasive procedures were varied, ranging between 80% and 90% with a specificity near 100% and a sensitivity between 66% and 90% [9,10,11]. Nowadays, robust data from ASTER trial [12] and MEDIASTrial ([13]) shows that invasive surgical staging can be omitted after negative systematic endosonography of mediastinum. Current indications for endosonographic systematic mediastinal staging in non-metastatic NSCLC include [14]: (1) presence of hilar and/or mediastinal lymphadenopathy suspicious due to CT criteria (short axis > 1 cm) and/or 18FDG-PET positive; (2) negative hilar-mediastinal lymph nodes based on CT/PET criteria in cases of central tumor, peripheral tumor > 3 cm, or poor/absent uptake of the primary tumor on PET. Consequently, invasive mediastinal staging is not required for a peripheral tumor < 3 cm with 18FDG-PET positive and hilar-mediastinal lymph nodes negative according to CT/PET-FDG criteria.

### 3.6. EUS—Elastography and Contrast Dye Evaluation

**Figure 5:**
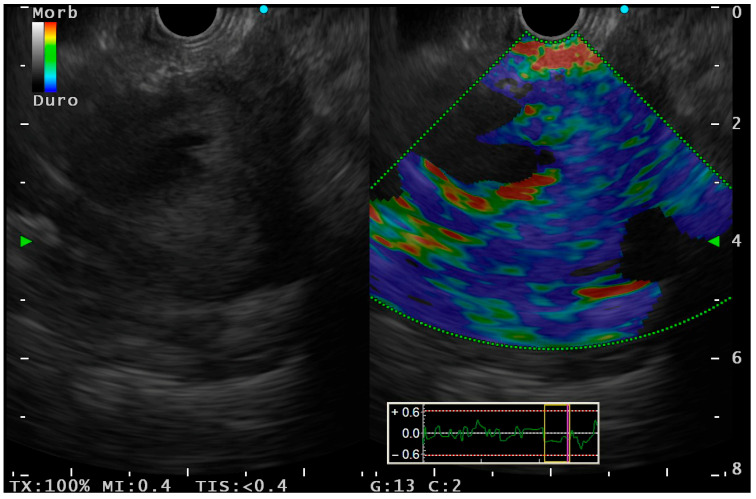
EUS view of elastography (right side) for the evaluation of lung cancer.

To improve the diagnostic yield of B-mode EUS, EUS-guided strain elastography (EUS-E), and contrast-enhanced harmonic EUS (CH-EUS) serve as useful adjuncts to tissue sampling by increasing its negative predictive value. However, both come with certain limitations. Only about 25% of mediastinal LNs can be confidently categorized as benign or malignant based solely on EUS feature [5]. EUS-E (Figure 5) is a non-invasive technique that assesses tissue elasticity in real time by detecting image changes in response to slight compression with the ultrasound probe. The principle underlying elastography is that different pathological processes, such as inflammation, fibrosis, and malignancy, induce distinct alterations in tissue stiffness. Elastographic deformation can be assessed qualitatively through color map distribution or quantitatively by evaluating strain ratios and histograms. Since 2019, shear wave elastography guided by EUS has been available, offering numerical values based on the velocity of shear wave propagation. Higher speeds typically indicate increased stiffness, suggesting malignancy [15,16].

In addition, CH-EUS represents another method for improving EUS-based differential diagnosis. The development of microbubble-based contrast agents, in conjunction with technological advancements, has significantly enhanced the visualization of fine vascular structures and microvascular patterns within target lesions [17,18]. CH-EUS combines a very high-resolution method, with the contrast-enhancing of the LNs’ capillary bed. Working on the assumption that the capillary bed of a metastatic tissue is destroyed, the contrast should be less visible [19]. Both EUS-based methods have demonstrated the ability to differentiate benign from malignant solid pancreatic masses, pancreatic cysts, and lymph nodes with high accuracy [20]. In the context of lung cancer, EUS-E has shown favorable sensitivity (89.4%) and a high negative predictive value (95.1%) for mediastinal LN staging. These diagnostic metrics are particularly notable when LNs exceed 1 cm in size on ultrasound [19]. Elastography may assist in identifying the most suspicious LN for biopsy, or in targeting the most concerning regions within a LN for sampling [19].

A recent meta-analysis reported that CH-EUS achieved a pooled sensitivity of 87.7% and specificity of 91.8%, yielding an overall diagnostic accuracy comparable to that of elastography and EUS-guided fine needle aspiration (FNA) [21]. These findings support the potential utility of CH-EUS, either alone or in combination with elastography in the evaluation and staging of LNs in patients with lung cancer. Both methods are promising tools for noninvasively characterizing LNs and may be especially valuable when added to the EUS-TA after a previous nondiagnostic FNA, or even as a guide before tissue sampling. However, its utility is constrained by variability between operators and patients’ limitations, absence of standardized protocols and cut-offs, need for special equipment not available everywhere and dependence on LN size, location (less accurate in deep LN and histological type) [19,21].

### 3.7. EUS Guided Tissue Acquisition

TA techniques under EUS guidance include FNA, cytological assessment, and fine needle biopsy (FNB) histological assessment. Common indications for EUS-TA include diagnosis and staging of pancreatobiliary and gastrointestinal malignancies, assessment of lymphadenopathy associated with luminal gastrointestinal cancers (esophagus, stomach, rectum), and lung cancer. The technique is also valuable for evaluating potentially neoplastic subepithelial gastrointestinal lesions and for assessing metastases in the liver, adrenal glands, or within the pleural or peritoneal cavities [22]. EUS-TA is a minimally invasive, safe procedure that can be performed with conscious sedation, making it suitable for outpatient settings, even if tertiary centers should take care of these patients in order to be able to act immediately in case of adverse events [23].

According to the European Society of Gastrointestinal Endoscopy (ESGE) Guidelines, both 25 G and 22 G needles are recommended for routine EUS-guided sampling of solid lesions and lymph nodes, with no preference between FNA and FNB. However, when the primary objective is to obtain core tissue for histological analysis, the use of 19 G FNA or FNB needles, or alternatively 22 G FNB needles, is advised. For LN sampling, the ESGE recommends using a 10 mL syringe for suction with 25 G or 22 G needles, ensuring that any negative pressure is neutralized before withdrawing the needle from the lesion. Moreover, the “slow-pull” technique, involving gradual withdrawal of the stylet to generate low negative pressure, may also be used because it has been associated with reduced blood contamination, although randomized trials show no clear superiority over standard suction. Additionally, the “fanning technique” involves redirecting the needle within the lymph node using the endoscope elevator to access multiple regions of the target tissue [24].

A range of FNA and FNB needles are available for EUS-guided LN sampling, each with distinct strengths and limitations. FNA needles, typically offered in 25 G, 22 G, and 19 G sizes, often feature a lancet tip with echogenic enhancement to improve visualization. The 19 G nitinol variants are especially flexible and well-suited for cytological assessment, though their ability to procure core tissue for histological analysis is limited [19]. In contrast, FNB needles are specifically designed for histology, with newer designs, such as the Franseen-tip, fork-tip, and reverse-bevel tip, providing improved tissue integrity and reduced fragmentation [25]. While recent meta-analyses suggest that EUS-FNA and EUS-FNB perform similarly in LNs sampling overall, end-cutting FNB needles have demonstrated superior diagnostic accuracy [26,27]. Overall, needle selection should be tailored to procedural goals, anatomical access, and the operator’s experience. Rapid On-Site Evaluation (ROSE) refers to the immediate cytological assessment of material obtained during FNA, and it is performed in the endoscopy room by a cytopathologist. Its purpose is to confirm the presence of diagnostically adequate material in real time, to enhance procedural efficiency by reducing the number of needle passes and to minimize the likelihood of repeat procedures due to non-diagnostic samples. According to ESGE Guidelines, EUS-guided sampling may be performed with or without ROSE, as both approaches are considered acceptable [28]. However, ROSE is not widely accessible due to the need for specialized personnel and increased operational costs [24]. In the absence of ROSE, ESGE recommends a standardized approach with multiple passes, typically three to four for FNA and two to three for FNB, to optimize diagnostic yield [24].

However, the main European societies suggested EUS in patients with a centrally located lung lesion that is not approachable at conventional bronchoscopy [4], or when bronchoscopy or EBUS resulted nondiagnostic. Surely, these lesions need to be reachable by transesophageal EUS approach. Moreover, EUS-TA for lung masses is mainly performed by transbronchial FNA-related techniques, showing a pooled adequacy of 95.4% and accuracy of 93.4% in a meta-analysis in which the included studies principally performed EUS-FNA [29]. Anyway, advances in needle technology, such as cryobiopsy needles for EBUS and third-generation needles for EUS, have further improved tissue sampling quality, especially in the case of EUS-FNB, which showed high accuracy (87.5%), sensitivity (97.92%) and specificity (100%) [30]. Mentioned EBUS-guided cryobiopsy has shown superior diagnostic performance in several settings. It outperforms transbronchial needle aspiration (EBUS TBNA) in diagnosing benign conditions such as sarcoidosis and pneumoconiosis. In malignant disease, cryobiopsy provides a higher diagnostic yield across both common and rare lung tumors and offers greater tissue adequacy for molecular testing, with reported success rates of around 95%. It also improves detection and characterization of lymphomas, achieving diagnostic yields exceeding 90%, compared to the approximately 77% sensitivity typically reported for EBUS TBNA. These findings suggest cryobiopsy may be particularly valuable when histological detail or molecular analysis is critical for diagnosis and management [31].

### 3.8. Clinical Use in Different Settings

Thoracic EUS is currently utilized for cancer staging, the assessment of LN enlargement, and the evaluation of subepithelial gastrointestinal lesions [32]. The literature also reports its use in the drainage of mediastinal fluid collections and abscesses [33]. Accurate staging is essential for determining the appropriate oncologic management. In esophageal cancer, although several imaging modalities are available, EUS is particularly valuable due to its ability to visualize the distinct layers of the esophageal wall and assess tumor depth and regional LNs involvement [34]. For lung cancer, no single technique offers complete access to all mediastinal LNs stations, but the combination of both EUS and EBUS permits access to all of them. Histological confirmation of nodal involvement is generally advised, except in cases with evident bulky disease. Minimally invasive approaches such as EBUS and EUS-guided tissue sampling, performed by experienced operators, are often preferred over mediastinoscopy due to their lower risk and cost [35]. The diagnostic yield improves when EUS-FNA is combined with EBUS-TBNA, as supported by multiple studies showing increased sensitivity in staging lung cancer [36]. Therefore, a combination of EUS-FNA and EBUS-FNA seems to increase TA performances to 86% of pooled sensitivity and 100% of pooled specificity in a meta-analysis for evaluating LNs staging [37]. Furthermore, EUS is a choice especially in those patients unfit for bronchoscopy [38]. For non-malignant conditions such as sarcoidosis, diagnosis relies on a combination of clinical, radiologic, and laboratory findings, occasionally supported by histology. PET imaging is also useful, and EUS or EBUS helps exclude malignancy-related lymphadenopathy [39]. Of note, in a 2022 multicenter randomized trial by Crombag et al., 358 patients with suspected stage I/II sarcoidosis were enrolled, with 185 assigned to EBUS-TBNA and 173 to EUS-B-FNA. Sarcoidosis was confirmed in 86% of cases. The detection rates and sensitivities based on granuloma identification were 70% and 78% for EBUS-TBNA, and 68% and 82% for EUS-B-FNA, respectively, with no significant difference between groups [40]. It seems that both techniques are a lucky combination according to Filarecka et al. who assessed the sequential use of EBUS-TBNA followed by EUS-B-FNA in 50 patients with suspected stage I/II sarcoidosis. Reported sensitivities were 76.6% for EBUS-TBNA, 70.2% for EUS-B-FNA, and 91.7% for the combined techniques [41]. In cases of suspected lymphoma, particularly in patients with prior disease, the integration of EUS and EBUS can be informative. Direct comparisons between EBUS and EUS for lymphoma diagnosis are lacking. In patients with extrapulmonary malignancies, including lymphoma (33%), EBUS-FNA achieved a sensitivity of 90.9% for PET-avid LNs, outperforming PET alone (72.7%) in identifying true-positive cases [42]. EUS-FNA/B, especially when combined with ancillary techniques such as immunocytochemistry and flow cytometry, has shown utility in diagnosing and subclassifying gastrointestinal lymphomas, with concordance to the WHO classification in 72.5% of cases [38]. For malignant lymphoma, EUS-FNB demonstrated superior diagnostic accuracy (75% vs. 50%) and sensitivity for histological diagnosis (100% vs. 78.9%) compared to FNA. It also provided higher WHO subclassification rates (71.4% vs. 31.6%) and better histological quality, with minimal adverse events reported [43].

Additionally, infectious diseases like tuberculosis or fungal infections can be diagnosed through samples obtained via EUS or EBUS, which should undergo appropriate microbiological staining and culture when systemic infection is suspected [44]. Also, EBUS/EUS-B has demonstrated high diagnostic performance for mediastinal tuberculosis in children, with reported sensitivity and negative predictive value of 100%, and specificity of 82.9% [45]. It is fundamental to be able to distinguish malignant from tuberculous LNs. Retrospective analysis showed that findings like LN conglomeration, coagulation necrosis, and distinct margins were more common in tuberculosis, while malignancies were associated with larger LNs and the presence of central intranodal vessels [46].

However, artificial intelligence (AI) holds promise for enhancing EUS by reducing the learning curve, improving diagnostic accuracy, and minimizing repeat procedures due to non-diagnostic biopsies [47]. In lymph node assessment, a recently published ensemble AI model achieved 80.6% overall accuracy, with high specificity (96.9%) and positive predictive value (85.9%) for detecting malignancy, despite this, sensitivity remained limited at 43.2% [48].

## 4. Conclusions

In conclusion, EUS is a fundamental tool for the diagnosis and staging of mediastinal lesions, but also for pulmonary lesions, permitting TA for histological examination. Its accuracy, combined with other approaches like EBUS, delivers high-quality diagnostic results, significantly impacting oncological treatment and even the management of non-oncological diseases in specific cases. The combination of transesophageal EUS and EBUS lead to the visualization of the majority of mediastinal LNs. In addition, the integration of innovative techniques such as elastography and contrast-enhanced imaging continues to expand EUS’s potential, improving tissue and lesion characterization. The possibility to use the samples acquired by these techniques for molecular tests permits oncological research, tailoring treatment options to every single patient [47,48].

## Figures and Tables

**Table 1:** Lymph node stations evaluated with EBUS depending on the area.

Zone	Station	Lymph Nodes
Supraclavicular Zone	1R and 1L	Lower cervical, supraclavicular, and jugular notch lymph nodes
Superior Zone	2R and 2L	Upper paratracheal lymph nodes
	3A	Prevascular lymph nodes
	3P	Retrotracheal lymph nodes
	4R and 4L	Lower paratracheal lymph nodes
Aorto-pulmonary Zone	5	Subaortic or aorto-pulmonary window lymph nodes
	6	Para-aortic lymph nodes
Subcarinal Zone	7	Subcarinal lymph nodes
Inferior Zone	8R and 8L	Paraesophageal lymph nodes
	9	Pulmonary ligament lymph nodes
Hilar and Interlobar Zone	10R and 10L	Hilar lymph nodes
	11R and 11L	Interlobar lymph nodes
Peripheral Zone	12R and 12L	Lobar lymph nodes
	13R and 13L	Segmental lymph nodes
	14R and 14L	Subsegmental lymph nodes

## Abbreviations

EUS	Endoscopic ultrasound
EBUS	Endobronchial ultrasound
LNs	Lymph Nodes
TA	Tissue acquisition
TBNA	Transbronchial needle aspiration
NSCLC	Non-Small Cell Lung Cancer
EUS-E	EUS-guided strain elastography
CH-EUS	Contrast-enhanced harmonic EUS
FNA	Fine needle aspiration
FNB	Fine needle biopsy
ROSE	Rapid On-Site Evaluation
